# Checklist das categorias da CIF relevantes para o desenvolvimento de fala e linguagem

**DOI:** 10.1590/2317-1782/20232022322en

**Published:** 2024-01-22

**Authors:** Fernanda Chequer de Alcântara Pinto, Ana Maria Schiefer, Jacy Perissinoto

**Affiliations:** 1 Programa de Pós-graduação em Distúrbios da Comunicação Humana, Escola Paulista de Medicina, Universidade Federal de São Paulo – UNIFESP - São Paulo (SP), Brasil.; 2 Departamento de Fonoaudiologia, Escola Paulista de Medicina, Universidade Federal de São Paulo – UNIFESP - São Paulo (SP), Brasil.

**Keywords:** Classificação Internacional de Funcionalidade, Incapacidade e Saúde, Fonoaudiologia, Classificações em Saúde, Linguagem Infantil, Desenvolvimento Infantil

## Abstract

**Objetivo:**

Criar um checklist da Classificação Internacional de Funcionalidade, Incapacidade e Saúde (CIF) a partir de categorias relevantes para o desenvolvimento de fala e linguagem, segundo a percepção de pais e fonoaudiólogos.

**Método:**

Realizou-se aplicação piloto e pesquisa. Na pesquisa participaram 100 pais de pré-escolares, com desenvolvimento típico de linguagem/cognição e 57 fonoaudiólogos especialistas em linguagem. Elaborou-se questionário com 199 categorias da CIF dos componentes de funções do corpo, atividades e participação e fatores ambientais. Cada categoria foi pontuada como: indispensável (2); importante (1) ou sem importância (0). Realizou-se a análise estatística (descritiva, soma, cluster/Método K-means e Mann-Whitney).

**Resultados:**

Com a soma dos pontos (indispensável, importante e sem importância) e a quantidade de respostas indispensável, foram identificadas as categorias de maior relevância para cada grupo de respondentes, assim como o conjunto de categorias em comum (72 consideradas de maior influência). A listagem comum aos grupos contou com os três componentes: funções do corpo (30 categorias/40% do total), atividades e participação (35/49,29%) e fatores ambientais (sete/13,20%). Das categorias selecionadas, 58,33% apresentaram resultados estatisticamente significantes entre os grupos, quanto à relevância dada.

**Conclusão:**

As categorias foram consideradas com pontuações distintas entre os grupos: as de funções do corpo foram mais pontuadas pelos fonoaudiólogos, enquanto as de fatores ambientais pelos pais. Assim, foi possível criar um checklist a partir da identificação das categorias mais relevantes para o desenvolvimento de fala e linguagem, em idade pré-escolar, contemplando os componentes funções do corpo, atividades e participação e fatores ambientais.

## INTRODUÇÃO

O desenvolvimento infantil e especificamente o de linguagem, resulta dos aspectos biopsicossociais, em seus componentes neurobiológicos, experienciais e circunstanciais^(1)^. Assim, como recurso, tem-se a valorização de procedimentos que considerem a percepção dos responsáveis, ampliando a visão sobre linguagem e funcionalidade^(2)^.

No acompanhamento da linguagem infantil, deve-se pensar em instrumentos que objetivem o mapeamento e rastreio do desenvolvimento, a partir de referenciais de normalidade e seus desvios. Para tanto, a Classificação Internacional de Funcionalidade, Incapacidade e Saúde (CIF) da Organização Mundial de Saúde^(3)^ pode auxiliar o fonoaudiólogo em atuações clínicas e de pesquisa na avaliação, orientação e intervenção individualizados, pela proposição abrangente de classificação em diferentes domínios do comportamento^(4)^.

Consequentemente, tanto na pesquisa quanto na clínica, a aplicação da CIF tem sido facilitada por meio de ferramentas constituídas e construídas na diversidade de percepções sobre a funcionalidade^(5)^. Sua estrutura organiza informações, sendo utilizada como ferramenta estatística e epidemiológica^(6)^ e no estudo da funcionalidade em pessoas saudáveis^(7)^.

As possibilidades de adoção da CIF permitem a criação de novos instrumentos, como entrevistas e questionários^(8,9)^. Em especial, viabilizam o reconhecimento de diferentes pontos de vista sobre um mesmo tópico, considerando os aspectos relevantes para o próprio usuário^(10)^ e para os responsáveis^(11)^. A organização da CIF permite que o profissional expanda sua visão sobre o desenvolvimento infantil, incluindo as diferentes expectativas e metas quanto ao desempenho comunicativo daquela criança frente ao contexto^(12)^.

Nesse cenário, cabe aos profissionais da saúde desenvolverem ferramentas e ações pautadas na CIF que estimulem a aproximação, praticidade e confiabilidade no processo de cuidar^(9,13)^ e que mostrem a relação entre as categorias de atividades e participação, habilidades de comunicação e fatores ambientais^(14)^, sendo também relativos ao desenvolvimento infantil^(15)^ e suas práticas de avaliação^(16)^.

Portanto, o objetivo deste estudo foi criar um checklist da Classificação Internacional de Funcionalidade, Incapacidade e Saúde (CIF) a partir de categorias relevantes para o desenvolvimento de fala e linguagem, segundo a percepção de pais e fonoaudiólogos. A seleção de determinadas categorias da CIF relacionadas à fala e linguagem, facilitará o entendimento das múltiplas influências no desenvolvimento e desempenho da comunicação para os processos de prevenção, avaliação e intervenção, facilitando a abrangência do olhar e a comunicação da equipe multidisciplinar.

## MÉTODO

Estudo observacional, analítico e transversal aprovado pelo Comitê de Ética em Pesquisa (CEP), sob protocolo 1.681.979/2016. Todos os participantes e instituições assinaram o Termo de Consentimento Livre e Esclarecido. Inicialmente foi realizada a elaboração de um questionário com categorias pré-selecionadas da CIF e posteriormente o instrumento foi aplicado.

### Participantes

A partir da realização de um cálculo pré-amostral, com uma amostragem por conveniência, foram entrevistados 100 pais (85 mães e 15 pais; média de 33 anos; 74% com ensino médio e 26% superior) de crianças (60 meninos e 40 meninas), com um média de 5,16 anos (idade mínima de quatro anos e máxima de cinco anos e onze meses), destes 37% entre quatro e quatros anos e onze meses e 63% entre cinco e cinco anos e onze meses. Quanto aos fonoaudiólogos (especialistas em Linguagem pelo Conselho Federal de Fonoaudiologia), a amostra contou com 57 profissionais (98,2% mulheres e média de 48,61 anos).

Os critérios de inclusão dos pais abarcavam: mais que oito anos de escolaridade, ter criança na faixa etária pré-escolar (entre quatro e cinco anos e onze meses), não apresentar queixa quanto ao desenvolvimento e não ter filho (a) com desempenho alterado nas avaliações (fonoaudiológica e neuropsicológica) realizadas. O critério de exclusão englobava respostas em branco no questionário aplicado.

Os critérios de inclusão dos fonoaudiólogos foram: atuar na cidade de São Paulo e ter o Título de Especialista em Linguagem pelo Conselho Federal de Fonoaudiologia vigente. Em relação aos de exclusão: restrição na atuação com a faixa etária em questão e qualquer ausência de respostas às perguntas do questionário.

### Procedimentos

Realizou-se a elaboração de um questionário visando identificar percepções acerca do desenvolvimento típico de fala e linguagem em pré-escolares, com categorias a serem investigadas e cuidadas pelos profissionais de saúde, garantido orientação e intervenção precoces. Justifica-se a escolha da faixa etária, pela importância de considerar o desenvolvimento infantil como um processo contínuo, a ser garantida sua adequada vigilância e busca de ações clínicas padronizadas e sensíveis na identificação de riscos e alterações.

Utilizou-se a Classificação Internacional de Funcionalidade, Incapacidade e Saúde (CIF)^(3)^ por oferecer um modelo biopsicossocial, estrutural, para uso na saúde e em áreas relacionadas, que leva em consideração os componentes de funções e estruturas do corpo, atividades e participação, fatores ambientais e pessoais. O sistema de classificação é hierárquico, dividido em capítulos e categorias de dois a quatro níveis^(3)^. A Classificação não é um instrumento avaliativo, porém permite a seleção de códigos que especificam a magnitude da funcionalidade ou incapacidade, ou em que medida um fator ambiental facilita ou constitui uma barreira.

A elaboração das questões abarcou categorias de três componentes da CIF: funções do corpo, atividades e participação e fatores ambientais. O componente de estrutura do corpo não foi incluído, pois considerou-se a preservação anatômica em pesquisa sobre normalidade^(17)^. Como indicado para pesquisas e avaliação de tratamento clínicos^(3)^ utilizou-se a subdivisão de segundo nível da CIF (362 categorias ao todo).

Foram retiradas as categorias não esperadas para a faixa etária e outras que preconizam a definição de situações não descritas pela CIF, chegando-se a 199 categorias que foram transformadas em perguntas e estruturadas em blocos de questões fechadas, de maneira que o respondente indicava o seu posicionamento. Adotou-se o formato de três itens escalonados^(18)^ com as alternativas: Indispensável (IN) – quando há concordância dominante – dois pontos; Importante (I) – quando há concordância predominante porém, nem todas as vezes – um ponto; Sem importância (SI) – quando há discordância predominante – zero ponto.

Como respondentes, selecionou-se grupos de pais de crianças e fonoaudiólogos, considerando a indicação da Organização Mundial de Saúde quanto a importância do envolvimento do indivíduo, por motivos éticos e pela construção dos achados com maior validade^(3)^.

Previamente, para a verificação do questionário, ocorreu a aplicação em dois grupos^(19)^ de caracterização semelhante aos grupos amostrais. Nesse contexto, cada indivíduo foi orientado a julgar a linguagem e o instrumento utilizado. Ressalta-se que não ocorreram modificações após o procedimento.

Para o estudo, dois grupos responderam ao questionário elaborado com 199 perguntas. Na aplicação no grupo de profissionais, as categorias foram apresentadas com a mesma descrição da CIF e eles deveriam responder a importância de cada uma delas para desenvolver a fala e a linguagem em idade pré-escolar. Com relação ao grupo de pais, optou-se pela abreviação e objetivação da descrição das categorias da CIF, de forma a não perder a sua definição e facilitando a compreensão. Cada indivíduo foi orientado a refletir sobre a sua criança em idade pré-escolar e responder o quão fundamental aquela categoria era para desenvolver adequadamente a fala e a linguagem.

A coleta do estudo foi realizada em uma escola de educação infantil, localizada na zona sul da cidade de São Paulo. Para participar, os pais responderam a uma pesquisa inicial sobre o desenvolvimento da criança. Posteriormente, o processo de avaliação constou da aplicação de uma triagem auditiva, a avaliação da linguagem receptiva e expressiva e um teste neuropsicológico (com auxílio de uma psicóloga), para medição do quociente de inteligência não verbal. Todos realizados no próprio ambiente escolar.

No processo de avaliação foi pesquisado o Reflexo cócleo palpebral (RCP), no qual foi apresentado para a criança um estímulo sonoro de alta intensidade e curta duração, que oferece dados do desenvolvimento maturacional global da criança. Dos aspectos de linguagem, utilizou-se a Avaliação do Desenvolvimento da Linguagem (ADL)^(20)^, escala que possui como objetivo avaliar os domínios receptivos e expressivos da linguagem, na faixa etária de um ano a seis anos e onze meses. Da avaliação neuropsicológica, mediu-se o quociente de inteligência (QI) não verbal, por meio do Son-R 2 ½-7 [a]^(21)^, que engloba os subtestes: mosaicos, padrões, categorias e situações, formando uma escala de execução e de raciocínio.

Do total de crianças matriculadas na escola, 134 (referente a 86,54% do total de escolares da instituição) receberam a autorização dos pais para serem avaliadas. Destas, 117 crianças apresentaram resultados adequados nos testes (87,31%) e 17 delas apresentaram resultados alterados, sendo excluídas da amostra e encaminhadas para serviço diagnóstico (12,69%).

Após a avaliação das crianças, grupos de até dez pais eram chamados por vez para realização da devolutiva da avaliação (até uma hora de duração). Durante o encontro e após breve explicação sobre a CIF, os pais eram orientados a responder o questionário, pontuando o grau de influência de cada categoria para o desenvolvimento de fala e linguagem em seus filhos. 106 pais responderam ao questionário e desse total seis foram excluídos de maneira aleatória para atingir os 100 pré-definidos.

Quanto ao grupo de fonoaudiólogos, optou-se por aqueles com especialização em linguagem atuantes na cidade de São Paulo e título válido de especialista pelo Conselho Federal de Fonoaudiologia (CFFa). Cada fonoaudiólogo permaneceu com o questionário, que continha breve explicação sobre a CIF, individualmente, por um prazo máximo de noventa dias.

### Análise estatística

Os achados foram tabulados e as respostas analisadas. Para verificar quais questões teriam maior relevância, calculou-se: a soma dos escores, correspondente aos valores zero, um ou dois, pontuados por pergunta – caracterizando o critério de análise SOMA; e realizou-se a contagem das respostas Indispensável, critério TOTAL2. No estabelecimento da classificação, fez-se uma análise por cluster (método K-means) por critério (SOMA e TOTAL2) e grupo (profissionais e pais), definindo-se cinco agrupamentos em ordem decrescente: 1 (maior pontuação), 2, 3, 4 e 5 (menor pontuação). Para comparação de respostas entre os grupos utilizou-se o teste não paramétrico de Mann-Whitney, com um nível de significância de 5% e um intervalo de confiança de 95%.

## RESULTADOS

Ressalta-se que todas as categorias investigadas receberam pontuações, o que confirma que não ocorreram ausências de respostas. Entre as dez categorias mais bem pontuadas, os pais consideraram Família nuclear (e310), do componente de fatores ambientais, como a mais relevante para o desenvolvimento de fala e linguagem. Para os fonoaudiólogos, a primeira categoria foi do componente de atividades e participação, Aquisição de linguagem (d132).

No grupo de pais, a categoria de atividades e participação que apareceu entre as dez primeiras foi Ouvir (d115), as demais estiveram relacionadas ao componente de funções do corpo, principalmente quanto à função mental, sistema cardiovascular e respiratório. Para os fonoaudiólogos, as categorias mais relevantes se dividiram entre funções do corpo, principalmente em seus capítulos de função mental, de voz e fala; e atividades e participação, quanto ao aprendizado e aplicação de conhecimento.

A construção do cluster se deu a partir dos resultados identificados pelos critérios SOMA e TOTAL2, identificando as categorias mais bem pontuadas para constituição do checklist de desenvolvimento típico de fala e linguagem. A Tabela 1 compara a seleção de categorias, entre as mais relevantes por grupo, a partir dos três primeiros agrupamentos. De acordo com os critérios previamente definidos, optou-se por fazer as exclusões das questões dos dois últimos agrupamentos (4 e 5), referentes às categorias com as menores pontuações definidas pelos grupos.

**Tabela 1:** Seleção cluster final para ambos os grupos

**Questão/ categoria**	**Cluster pais**	**Cluster fono**	**Checklist final**
Funções da consciência (b110)	2	1	1
Funções da orientação (b114)	2	1	1
Funções intelectuais (b117)	1	1	1
Funções psicossociais globais (b122)	2	1	1
Funções do temperamento e da personalidade (b126)	3	3	1
Funções da energia e de impulsos (b130)	3	3	1
Funções do sono (b134)	3	3	1
Funções da atenção (b140)	2	1	1
Funções da memória (b144)	2	1	1
Funções psicomotoras (b147)	2	2	1
Funções emocionais (b152)	3	2	1
Funções da percepção (b156)	1	1	1
Funções do pensamento (b160)	2	2	1
Funções cognitivas superiores (b164)	3	1	1
Funções mentais da linguagem (b167)	2	1	1
Funções mentais de sequenciamento de movimentos complexos (b176)	3	2	1
Funções de experiência pessoal e de tempo (b180)	3	2	1
Funções da visão (b210)	2	3	1
Funções auditivas (b230)	2	1	1
Funções vestibulares (b235)	3	3	1
Função proprioceptiva (b260)	3	3	1
Funções da voz (b310)	2	1	1
Funções da articulação (b320)	2	1	1
Funções da fluência e do ritmo da fala (b330)	2	1	1
Funções alternativas de vocalização (b340)	3	2	1
Funções respiratórias (b440)	1	3	1
Funções dos músculos respiratórios (b445)	1	3	1
Funções de ingestão (b510)	1	3	1
Funções relacionadas à força muscular (b730)	3	3	1
Funções relacionadas ao controle dos movimentos voluntários (b760)	3	3	1
Observar (d110)	2	3	1
Ouvir (d115)	1	1	1
Outras percepções sensoriais intencionais (d120)	2	3	1
Imitar (d130)	3	1	1
Aquisição de linguagem (d132)	3	1	1
Ensaiar (d135)	3	2	1
Aquisição de conceitos (d137)	3	1	1
Aquisição de informação (d138)	2	1	1
Aprender a ler (d140)	2	3	1
Aprender a escrever (d145)	2	3	1
Aquisição de habilidades (d155)	3	2	1
Concentrar a atenção (d160)	2	2	1
Pensar (d163)	3	2	1
Tomar decisões (d177)	3	3	1
Realizar uma única tarefa (d210)	3	3	1
Realizar a rotina diária (d230)	3	3	1
Comunicação – recepção de mensagens orais (d310)	2	2	1
Comunicação – recepção de mensagens não-verbais (d315)	3	2	1
Comunicação–recepção de mensagens na linguagem de sinais convencionais (d320)	3	2	1
Fala (d330)	2	1	1
Cantar (d332)	2	2	1
Produção de mensagens não verbais (d335)	3	2	1
Conversação (d350)	3	1	1
Comer (d550)	2	3	1
Beber (d560)	2	3	1
Interações interpessoais básicas (d710)	3	2	1
Interações interpessoais complexas (d720)	3	3	1
Relações formais (d740)	2	3	1
Relações sociais informais (d750)	3	3	1
Relações familiares (d760)	2	2	1
Educação informal (d810)	3	3	1
Educação infantil (d815)	1	2	1
Educação escolar (d820)	1	2	1
Recreação e lazer (d920)	3	2	1
Direitos Humanos (d940)	3	3	1
Som (e250)	3	2	1
Família nuclear (e310)	1	2	1
Família ampliada (e315)	2	3	1
Amigos (e320)	3	3	1
Profissionais da saúde (e355)	2	3	1
Outros profissionais (e360)	3	3	1
Serviços, sistemas e políticas de saúde (e580)	2	3	1

Nota: Análise por cluster

O Quadro 1 indica o checklist que foi constituído a partir da indicação dos pais e profissionais sobre as categorias mais relevantes para o desenvolvimento de fala e linguagem a partir da CIF.

**Quadro 1:** Checklist da CIF para o desenvolvimento de fala e linguagem em pré-escolares

**CHECKLIST DA CIF PARA O DESENVOLVIMENTO DE FALA E LINGUAGEM EM PRÉ-ESCOLARES**
**Componente de funções do corpo**
Funções da consciência (b110) – funções mentais gerais do estado de alerta e de consciência, incluindo a clareza e continuidade do estado de vigília.
Funções da orientação (b114) – funções mentais gerais relacionadas ao conhecimento e determinação da relação da pessoa consigo própria, com outras pessoas, com objetos e com o espaço.
Funções intelectuais (b117) – funções mentais gerais, necessárias para compreender e integrar de forma construtiva as diferentes funções mentais, incluindo todas as funções cognitivas e seu desenvolvimento ao longo da vida.
Funções psicossociais globais (b122) – funções mentais gerais, como elas se desenvolvem ao longo da vida, necessárias para compreender e integrar construtivamente as funções mentais que levam à formação das habilidades interpessoais necessárias para o estabelecimento de interações sociais recíprocas, tanto em termos de significado como de objetivo.
Funções do temperamento e da personalidade (b126) – funções mentais gerais relacionadas com um temperamento que faz o indivíduo reagir de uma determinada maneira a situações, incluindo o conjunto de características mentais que diferenciam esse indivíduo das outras pessoas.
Funções da energia e de impulsos (b130) – funções mentais gerais dos mecanismos fisiológicos e psicológicos que estimulam o indivíduo a agir de modo persistente para satisfazer suas necessidades específicas e seus objetivos.
Funções do sono (b134) – funções mentais gerais de desconexão física e mental do ambiente imediato, de caráter periódico, reversível e seletivo, acompanhada por mudanças fisiológicas características.
Funções da atenção (b140) – funções mentais específicas de concentração em um estímulo externo ou experiência interna pelo período de tempo necessário.
Funções da memória (b144) – funções mentais específicas de registro e armazenamento de informações e sua recuperação quando necessário.
Funções psicomotoras (b147) – funções mentais específicas de controle dos eventos motores e psicológicos em nível corporal.
Funções emocionais (b152) – funções mentais específicas relacionadas ao sentimento e aos componentes afetivos dos processos mentais.
Funções da percepção (b156) – funções mentais específicas relacionadas com o reconhecimento e interpretação dos estímulos sensoriais.
Funções do pensamento (b160) – funções mentais específicas relacionadas ao componente ideativo da mente.
Funções cognitivas superiores (b164) – funções mentais específicas especialmente dependentes dos lobos frontais do cérebro, incluindo comportamentos complexos direcionados para metas, como tomada de decisão, pensamento abstrato, planejamento e execução de planos, flexibilidade mental e decisão sobre quais são os comportamentos adequados em circunstâncias específicas, chamadas com frequência de funções executivas.
Funções mentais da linguagem (b167) – funções mentais específicas de reconhecimento e utilização de sinais, símbolos e outros componentes de uma linguagem.
Funções mentais de sequenciamento de movimentos complexos (b176) – funções mentais específicas de sequenciamento e coordenação de movimentos complexos e com finalidade específica.
Funções de experiência pessoal e de tempo (b180) – funções mentais específicas relacionadas à consciência da própria identidade, do próprio corpo, de sua postura em seu ambiente e no tempo.
Funções da visão (b210) – funções sensoriais relacionadas com a percepção de luz e forma, tamanho, formato e cor de um estímulo visual.
Funções auditivas (b230) – funções sensoriais que permitem perceber sons e discriminar sua localização, intensidade, ruído e qualidade.
Funções vestibulares (b235) – funções sensoriais da orelha interna relacionadas à posição, ao equilíbrio e ao movimento.
Função proprioceptiva (b260) – funções sensoriais que permitem sentir a posição relativa das partes do corpo.
Funções da voz (b310) – funções da produção de vários sons pela passagem de ar através da laringe.
Funções da articulação (b320) – funções da produção de sons da fala.
Funções da fluência e do ritmo da fala (b330) – funções da produção do fluxo e ritmo da fala.
Funções alternativas de vocalização (b340) – funções da produção de outras formas de vocalização.
Funções respiratórias (b440) – funções relacionadas à inalação de ar para os pulmões, à troca de gases entre o ar e o sangue e à expulsão do ar.
Funções dos músculos respiratórios (b445) – funções dos músculos envolvidos na respiração.
Funções de ingestão (b510) – funções relacionadas à ingestão e manipulação de sólidos ou líquidos no corpo através da boca.
Funções relacionadas à força muscular (b730) – funções relacionadas à força gerada pela contração de um músculo ou grupo de músculos.
Funções relacionadas ao controle dos movimentos voluntários (b760) – funções relacionadas ao controle dos movimentos voluntários.
**Componente de atividades e participação**
Observar (d110) – utilizar intencionalmente o sentido da visão para experimentar estímulos visuais, como acompanhar ou seguir visualmente um objeto, assistir a um evento esportivo ou observar pessoas ou crianças brincando.
Ouvir (d115) – utilizar intencionalmente o sentido da audição para experimentar estímulos auditivos, como ouvir rádio, vozes humanas, música, uma aula ou o relato de uma história.
Outras percepções sensoriais intencionais (d120) – utilizar intencionalmente os outros sentidos básicos do corpo para experimentar estímulos, como tocar ou sentir texturas, saborear doces ou sentir o cheiro das flores.
Imitar (d131) – imitar ou espelhar como componente básico do aprendizado, como copiar uma expressão facial, um gesto, um som ou as letras de um alfabeto.
Aquisição de linguagem (d132) – desenvolver a capacidade de representar pessoas, objetos, acontecimentos, sentimentos por meio de palavras, símbolos, frases e sentenças.
Ensaiar (d135) – repetir uma sequência de eventos ou símbolos como componente básico do aprendizado, como contar de dez em dez ou praticar a recitação de uma rima com gestos ou acordes em um instrumento musical.
Aquisição de conceitos (d137) – desenvolver a capacidade de entender e usar conceitos básicos e complexos relacionados às características de coisas, pessoas ou acontecimentos.
Aquisição de informação (d138) – obter fatos sobre pessoas, coisas e acontecimentos, como perguntar por quê, o quê, onde e como, perguntar por nomes.
Aprender a ler (d140) – desenvolver a capacidade de ler material impresso (incluindo braile e outros símbolos) com fluência e precisão, como reconhecer caracteres e letras do alfabeto, vocalizar palavras escritas com a pronúncia correta e compreender palavras escritas de frases.
Aprender a escrever (d145) – desenvolver a capacidade de produzir símbolos que representam sons, palavras ou frases que tenham um significado (incluindo a escrita braile e outros símbolos), como escrever de maneira eficiente e utilizar a gramática correta.
Aquisição de habilidades (d155) – desenvolver capacidades básicas e complexas em usar conjunto integrado de ações ou tarefas de maneira a iniciar e concluir a aquisição de uma habilidade, como manipular ferramentas ou brinquedos, ou disputar jogos.
Concentrar a atenção (d160) – centrar-se intencionalmente em um estímulo específico, desligando-se de ruídos que distraem a atenção.
Pensar (d163) – formular e manipular ideias, conceitos e imagens, dirigidos ou não a um objetivo, sozinho ou com outros, como criar ficção, comprovar um teorema, brincar com ideias, debater ideias, meditar, ponderar, especular ou refletir.
Tomar decisões (d177) – fazer uma escolha entre opções, implementar a escolha e avaliar os efeitos da escolha, como selecionar e adquirir um item específico ou decidir pôr em prática e realizar uma tarefa entre várias tarefas que precisam ser feitas.
Realizar uma única tarefa (d210) – realizar ações simples ou complexas e coordenadas relacionadas aos componentes mentais e físicos de uma única tarefa, como iniciar uma tarefa, organizar o tempo, o espaço e os materiais para uma tarefa, regular o desempenho da tarefa e executar, concluir e manter a tarefa.
Realizar a rotina diária (d230) – realizar e coordenar ações simples ou complexas para planejar, gerenciar e concluir as exigências dos procedimentos ou dos deveres do dia a dia, como administrar o tempo e fazer planos para diversas atividades ao longo do dia.
Comunicação – recepção de mensagens orais (d310) – compreender os significados literal e implícito das mensagens em linguagem oral, como distinguir se uma frase tem um significado literal ou é uma expressão idiomática.
Comunicação – recepção de mensagens não-verbais (d315) – compreender os significados literal e implícito das mensagens transmitidas por gestos, símbolos e desenhos, como perceber que uma criança está cansada quando ela esfrega os olhos ou que um alarme significa que há incêndio.
Comunicação – recepção de mensagens na linguagem de sinais convencionais (d320) – receber e compreender mensagens na linguagem de sinais convencionais com significado literal e implícito.
Fala (d330) – produzir palavras, frases e passagens mais longas em mensagens faladas com significado literal e implícito, como expressar um fato ou contar uma história em linguagem oral.
Cantar (d332) – usar tons em uma sequência que resulte em melodia para transmitir mensagens.
Produção de mensagens não verbais (d335) – usar gestos, símbolos e desenhos para transmitir mensagens, como sacudir a cabeça para indicar desacordo ou desenhar uma figura ou um diagrama para transmitir um fator ou uma ideia complexa.
Conversação (d350) – iniciar, manter e finalizar uma troca de pensamentos e ideias, realizada por meio da linguagem escrita, oral, de sinais ou de outras formas de linguagem, com uma ou mais pessoas conhecidas ou estranhas, em um ambiente formal ou informal.
Comer (d550) – executar as tarefas e ações coordenadas de comer o alimento servido, levá-lo à boca e consumi-lo de maneira naturalmente aceitável; cortar ou partir o alimento em pedaços; abrir embalagens e pacotes; utilizar utensílios para comer; atividades relacionadas com refeições, banquetes e jantares.
Beber (d560) – pegar a bebida, levá-la à boca e consumir a bebida de maneira culturalmente aceitável, misturar, meses e verter líquidos para beber, abrir garrafas e latas, beber através de um canudo ou beber água corrente da torneira ou de uma fonte; amamentar.
Interações interpessoais básicas (d710) – interagir com as pessoas de maneira contextual e socialmente adequada, como mostrar consideração e estima quando apropriado ou reagir aos sentimentos dos outros.
Interações interpessoais complexas (d720) – manter e controlar as interações com outras pessoas, de maneira contextual e socialmente apropriada, como controlar emoções e impulsos, controlar a agressão verbal e física, agir de maneira independente nas interações sociais e agir de acordo com as regras e convenções sociais quando, por exemplo, estiver brincando, estudando ou trabalhando com outras pessoas.
Relações formais (d740) – criar e manter relações específicas em ambientes formais, como com professores, funcionários, profissionais ou prestadores de serviço.
Relações sociais informais (d750) – iniciar relações com os outros, como relações causais com pessoas que vivem na mesma comunidade ou residência, ou com colaboradores, estudantes, companheiros de lazer ou pessoas com formação ou profissão similares.
Relações familiares (d760) – criar e manter relações de parentesco, como com membros do núcleo familiar, parentes, família adotiva e de criação, e parentes não consanguíneos, relações mais distantes como primos de segundo grau ou tutores legais.
Educação informal (d810) – aprender em casa ou em outro ambiente não institucional, como aprender trabalhos manuais e outras habilidades como pais ou membros familiares, ou escolarização em casa.
Educação infantil (d815) – aprender em um nível inicial de instrução organizada, projetada principalmente para introduzir a criança no ambiente escolar e prepará-la para a educação obrigatória, como adquirir habilidades em uma creche ou em um ambiente similar em preparação para a escola.
Educação escolar (d820) – obter acesso à escola, participar de todas as responsabilidades e os privilégios relacionados à escola, e aprender o material do curso, a matéria e outras exigências curriculares em um programa educacional fundamental e médio, incluindo ir à escola regularmente, trabalhar em cooperação com outros alunos, seguir as orientações dos professores, organizar, estudar e concluir tarefas e projetos designados, e progredir para os outros estágios de educação.
Recreação e lazer (d920) – participar de qualquer forma de jogo, atividade recreativa ou de lazer, como jogo ou esportes informais ou organizados, programa de exercício físico, relaxamento, diversão, ir a galerias de arte, museus, cinema ou teatro, participar de trabalhos artesanais ou *hobbies*, ler por prazer, tocar instrumentos musicais, fazer excursões, turismo ou viajar por prazer.
Direitos Humanos (d940) – desfrutar de todos os direitos nacional e internacionalmente reconhecidos que são atribuídos às pessoas pelo simples fato de sua condição humana, como os direitos humanos reconhecidos pela Nações Unidas na Declaração Universal dos Direitos Humanos (1948), na Convenção sobre os Direitos da Criança (1989), nas Normas sobre Igualdade de Oportunidades para Pessoas com Deficiência (1993) e na Convenção sobre os Direitos das Pessoas com Deficiência (2006); o direito à autodeterminação ou autonomia; e o direito de controlar o próprio destino.
**Componente de fatores ambientais**
Som (e250) – um fenômeno que é ou pode ser ouvido, como batida, toque, pancada, canto, assobio, grito ou zumbido, em qualquer volume, timbre ou tom, e que pode fornecer informações úteis sobre o mundo.
Família nuclear (e310) – indivíduos relacionados por nascimento, casamento ou outros relacionamentos reconhecidos pela cultura, como família nuclear, cônjuges, parceiros, pais, irmãos, filhos, pais de criação, pais adotivos e avós.
Família ampliada (e315) – indivíduos aparentados por meio de família ou casamento ou outros relacionamentos reconhecidos pela cultura, como parentes, tias, tios, sobrinhos e sobrinhas.
Amigos (e320) – indivíduos que são próximos e contínuos em relacionamentos caracterizados por confiança e apoio mútuos.
Profissionais da saúde (e355) – todos os fornecedores de serviços que trabalham no contexto do sistema de saúde, como médicos, enfermeiros, fisioterapeutas, terapeutas ocupacionais, fonoaudiólogos, audiologistas, protéticos, assistentes sociais da área médica.
Outros profissionais (e360) – todos os fornecedores de serviço que trabalham fora do sistema de saúde, mas que fornecem serviços relacionados à saúde, como assistentes sociais, professores, arquitetos ou projetistas.
Serviços, sistemas e políticas de saúde (e580) – serviços, sistemas e políticas de prevenção e tratamento de problemas de saúde, fornecimento de reabilitação médica e promoção de um estilo de vida saudável.
**Componente de estruturas do corpo (algo a declarar):**
**Componente de fatores pessoais (descrição):**

Fonte: as autoras, 2023

O checklist definido nesta pesquisa contou com 72 categorias. Destas, quanto às funções do corpo, 30 estiveram presentes de acordo com a subdivisão por capítulos da CIF: 17 de funções mentais; quatro de funções sensoriais e dor; quatro de funções da voz e da fala; duas de funções dos sistemas cardiovascular, hematológico, imunológico e respiratório; uma de funções dos sistemas digestório, metabólico e endócrino; duas de funções neuromusculoesqueléticas e relacionadas ao movimento.

Quanto a atividades e participação, 35 categorias mostram-se de maior relevância: 14 do capítulo de aprendizagem e aplicação do conhecimento, duas de tarefas e demandas gerais, sete de comunicação, duas de cuidado pessoal, cinco de relações e interações interpessoais, três de áreas da vida e duas de vida comunitária, social e cívica.

Quanto aos fatores ambientais, sete categorias pertenciam ao componente, de três capítulos: ambiente natural e mudanças ambientais feitas pelo homem; apoio e relacionamentos; serviços, sistemas e políticas.

Na análise comparativa entre as respostas de ambos os grupos (Tabela 2), 42 categorias (58,33%) apresentaram-se com resultados significantes intergrupos, quanto a diferença na relevância dada. Destas 21 de funções do corpo (50%); 18 de atividades e participação (42,85%) e 3 de fatores ambientais (7,14%).

**Tabela 2:** Comparativo de respostas entre pais e fonoaudiólogos

**CATEGORIA**	**MÉDIA SOMA GRUPO PAIS**	**MÉDIA SOMA GRUPO FONOAUDIÓLOGOS**	**TESTE MANN-WHITNEY (p)**	**RESULTADO**
Funções da consciência (b110)	1,37	1,65	0,001*	Pais < Profissionais
Funções da orientação (b114)	1,32	1,61	0,001*	Pais < Profissionais
Funções intelectuais (b117)	1,54	1,74	0,015*	Pais < Profissionais
Funções psicossociais globais (b122)	1,32	1,77	<0,001*	Pais < Profissionais
Funções do temperamento e da personalidade (b126)	1,22	1,05	0,111	Pais = Profissionais
Funções da energia e de impulsos (b130)	1,22	1,04	0,080	Pais = Profissionais
Funções do sono (b134)	1,18	1,07	0,308	Pais = Profissionais
Funções da atenção (b140)	1,28	1,58	0,001*	Pais < Profissionais
Funções da memória (b144)	1,33	1,72	<0,001*	Pais < Profissionais
Funções psicomotoras (b147)	1,27	1,25	0,967	Pais = Profissionais
Funções emocionais (b152)	1,22	1,37	0,073	Pais = Profissionais
Funções da percepção (b156)	1,44	1,63	0,032*	Pais < Profissionais
Funções do pensamento (b160)	1,29	1,47	0,034*	Pais < Profissionais
Funções cognitivas superiores (b164)	1,20	1,61	<0,001*	Pais < Profissionais
Funções mentais da linguagem (b167)	1,34	1,82	<0,001*	Pais < Profissionais
Funções mentais de sequenciamento de movimentos complexos (b176)	1,18	1,21	0,600	Pais = Profissionais
Funções de experiência pessoal e de tempo (b180)	1,19	1,37	0,033*	Pais < Profissionais
Funções da visão (b210)	1,33	0,95	<0,001*	Pais < Profissionais
Funções auditivas (b230)	1,31	1,67	<0,001*	Pais < Profissionais
Funções vestibulares (b235)	1,17	1,00	0,076	Pais = Profissionais
Função proprioceptiva (b260)	1,18	1,16	0,969	Pais = Profissionais
Funções da voz (b310)	1,29	1,54	0,003*	Pais < Profissionais
Funções da articulação (b320)	1,30	1,74	<0,001*	Pais < Profissionais
Funções da fluência e do ritmo da fala (b330)	1,29	1,67	<0,001*	Pais < Profissionais
Funções alternativas de vocalização (b340)	1,16	1,33	0,029*	Pais < Profissionais
Funções respiratórias (b440)	1,48	1,12	0,001*	Pais > Profissionais
Funções dos músculos respiratórios (b445)	1,47	1,09	0,001*	Pais > Profissionais
Funções de ingestão (b510)	1,39	0,89	<0,001*	Pais > Profissionais
Funções relacionadas à força muscular (b730)	1,25	1,05	0,032*	Pais > Profissionais
Funções relacionadas ao controle dos movimentos voluntários (b760)	1,24	1,21	0,838	Pais = Profissionais
Observar (d110)	1,37	1,21	0,088	Pais = Profissionais
Ouvir (d115)	1,47	1,68	0,009*	Pais < Profissionais
Outras percepções sensoriais intencionais (d120)	1,33	1,02	0,001*	Pais > Profissionais
Imitar (d130)	1,21	1,68	<0,001*	Pais < Profissionais
Aquisição de linguagem (d132)	1,16	1,89	<0,001*	Pais < Profissionais
Ensaiar (d135)	1,19	1,42	0,007*	Pais < Profissionais
Aquisição de conceitos (d137)	1,17	1,68	<0,001*	Pais < Profissionais
Aquisição de informação (d138)	1,29	1,77	<0,001*	Pais < Profissionais
Aprender a ler (d140)	1,35	0,81	<0,001*	Pais > Profissionais
Aprender a escrever (d145)	1,34	0,79	<0,001*	Pais > Profissionais
Aquisição de habilidades (d155)	1,23	1,28	0,437	Pais = Profissionais
Concentrar a atenção (d160)	1,28	1,47	0,056	Pais = Profissionais
Pensar (d163)	1,21	1,49	0,003*	Pais < Profissionais
Tomar decisões (d177)	1,23	1,04	0,043*	Pais > Profissionais
Realizar uma única tarefa (d210)	1,18	1,05	0,182	Pais = Profissionais
Realizar a rotina diária (d230)	1,16	1,05	0,301	Pais = Profissionais
Comunicação – recepção de mensagens orais (d310)	1,29	1,39	0,228	Pais = Profissionais
Comunicação – recepção de mensagens não-verbais (d315)	1,23	1,39	0,053	Pais = Profissionais
Comunicação–recepção de mensagens na linguagem de sinais convencionais (d320)	1,14	1,14	0,738	Pais = Profissionais
Fala (d330)	1,33	1,60	0,004*	Pais < Profissionais
Cantar (d332)	1,28	1,33	0,528	Pais = Profissionais
Produção de mensagens não verbais (d335)	1,18	1,42	0,004*	Pais < Profissionais
Conversação (d350)	1,23	1,51	0,001*	Pais < Profissionais
Comer (d550)	1,26	0,88	0,001*	Pais > Profissionais
Beber (d560)	1,29	0,89	0,001*	Pais > Profissionais
Interações interpessoais básicas (d710)	1,25	1,42	0,039*	Pais > Profissionais
Interações interpessoais complexas (d720)	1,21	1,14	0,531	Pais > Profissionais
Relações formais (d740)	1,34	0,98	<0,001*	Pais > Profissionais
Relações sociais informais (d750)	1,16	1,05	0,276	Pais = Profissionais
Relações familiares (d760)	1,26	1,19	0,698	Pais > Profissionais
Educação informal (d810)	1,21	1,18	0,672	Pais > Profissionais
Educação infantil (d815)	1,41	1,35	0,658	Pais = Profissionais
Educação escolar (d820)	1,41	1,35	0,885	Pais = Profissionais
Recreação e lazer (d920)	1,25	1,23	0,998	Pais = Profissionais
Direitos Humanos (d940)	1,20	1,07	0,338	Pais = Profissionais
Som (e250)	1,14	1,26	0,114	Pais = Profissionais
Família nuclear (e310)	1,54	1,42	0,160	Pais = Profissionais
Família ampliada (e315)	1,26	1,05	0,032*	Pais > Profissionais
Amigos (e320)	1,21	1,23	0,929	Pais = Profissionais
Profissionais da saúde (e355)	1,31	1,16	0,172	Pais = Profissionais
Outros profissionais (e360)	1,21	0,96	0,025*	Pais > Profissionais
Serviços, sistemas e políticas de saúde (e580)	1,32	1,02	0,012*	Pais > Profissionais

Nota: Teste de Mann-Whitney

* P-valores com marcação (*) indicam um resultado estatisticamente significante

No que se refere ao componente de funções do corpo, observa-se que das 30 categorias analisadas do checklist, os fonoaudiólogos tiveram uma variável SOMA com maior pontuação que os pais em 18 (60%). Quanto às atividades e participação, das 35 categorias selecionadas, os fonoaudiólogos tiveram maior pontuação em 16 (45,71%). Por fim, no componente de fatores ambientais, observa-se que das sete categorias selecionadas, os fonoaudiólogos tiveram uma variável SOMA com maior pontuação que o grupo de pais em duas delas (28,57%).

## DISCUSSÃO

O propósito do estudo foi a criação de um checklist com base nos aspectos de funcionalidade em fala e linguagem. O uso desse instrumento visa facilitar a abrangência do olhar dos profissionais, possibilitando conhecer interações e impactos para que a criança adquira e desenvolva a fala e a linguagem. Ressalta-se que o uso da CIF como base estrutural, facilita a seleção e formalização de categorias, uniformizando a nomenclatura utilizada e possibilitando a comparação entre dados, confirmando a possibilidade de construção de outros instrumentos de avaliação, triagem e/ou acompanhamento do desenvolvimento com o seu escopo^(22)^.

Uma revisão sistemática analisando o uso da CIF na pediatria no Brasil, indicou que 40% dos estudos utilizaram checklists da Classificação em sua elaboração^(23)^ apesar de observarem um uso incipiente na área infantil por parte dos profissionais da saúde. Os autores reforçaram a importância do uso na definição e planejamento de recursos, serviços e políticas, organizando a informação, facilitando a comunicação e buscando abordagens mais holísticas.

A construção de instrumentos baseados na normalidade configura e abrange aspectos relevantes de acordo com a faixa etária e permite ao profissional uma visão ampliada da saúde, a identificação de comportamentos e situações que se distanciam da tipicidade. Tal processo facilita o processo de tomada de decisão, acompanhamento e vigilância infantil, assim como caracteriza de forma mais individualizada as demandas de funcionalidade da população. Porém, como reforçado por autores^(7,23)^, a pesquisa com populações sem alterações de saúde na lógica da prevenção e promoção utilizando a CIF, ainda se encontra em defasagem mesmo que a OMS preveja a inserção da Classificação nas populações, independentemente da presença de um quadro clínico^(3).^

O uso da CIF permite reconhecer as relações presentes entre os componentes dentro do desenvolvimento infantil, facilitando a investigação e permitindo que aspectos importantes da vivência social e interativa do sujeito sejam levados em consideração, para além do funcionamento do seu corpo. A importância de rastrear e monitorar indivíduos durante o percurso de desenvolvimento é reforçado por estudo anterior, que indicou o auxílio do uso da Classificação na organização e padronização das informações sobre funcionalidade. Os autores reforçaram que o desenvolvimento de instrumentos relacionados a CIF, como checklists, são fundamentais para a utilização do modelo biopsicossocial na prática clínica, em serviços que englobam triagem auditiva, acompanhamento do desenvolvimento de linguagem e evolução terapêutica^(8)^.

A utilização da estrutura da Classificação Internacional de Funcionalidade, Incapacidade e Saúde (CIF) é preconizada na prática clínica fonoaudiológica, com incentivo ao seu uso. Em uma revisão de escopo, a análise temática mostrou que a CIF foi aplicada na avaliação, intervenção e prestação de serviços de saúde. Porém, foi indicado que existem lacunas no uso de ferramentas baseadas na CIF na prática clínica fonoaudiológica e na sua compreensão social, cujo uso facilita uma compreensão holística da funcionalidade, incapacidade e decisões clínicas baseadas em evidências^(16)^.

Em uma revisão integrativa da literatura^(24)^ quanto ao uso da CIF, observou-se utilidade desde o processo de anamnese até o desenvolvimento da linguagem em crianças, incluindo o uso de questionários que simplifiquem a Classificação, assim como a criação de ferramentas que a partir de suas premissas, visem a aquisição e o desenvolvimento da linguagem. O que reforça a importância da construção de um checklist voltado para o desenvolvimento da linguagem infantil, como estruturado neste trabalho.

O desenvolvimento de competências de atuação fundamentado em uma ampla variedade de métodos de coleta de dados e ferramentas de documentação, ajuda os fonoaudiólogos a comunicarem com precisão os resultados dos serviços que prestam, aumentando assim a capacidade de tomar decisões clínicas^(25)^. O que impacta diretamente no entendimento da dimensão de atuação da área fonoaudiológica e a necessidade de maior foco nos aspectos de funcionalidade, principalmente relacionados à comunicação^(14)^.

Para delinear esses achados, a percepção dos profissionais com expertise na área se faz fundamental, assim como dos sujeitos, considerando que esses possuem o conhecimento quanto aos aspectos impactantes, de funcionalidade e incapacidade vivenciados. O uso da CIF possibilitou conhecer as categorias de relevância para o desenvolvimento típico de fala e linguagem, contemplando os componentes de funções do corpo, atividades e participação e fatores ambientais, assim como outros estudos que evidenciaram a aplicabilidade do uso da CIF como norteadores de serviços de saúde^(9,13,23,26)^.

Dentre as categorias mais relevantes pontuadas pelos grupos, estiveram presentes principalmente aquelas relacionadas ao componente de função do corpo, o que está de acordo com outros estudos^(27)^. Tais achados demonstram que os aspectos fisiológicos são considerados como de grande relevância quando se pensa no desenvolvimento da criança, o que fortalece a estrutura de formação do profissional e de divulgação do conhecimento da saúde constituído no modelo biomédico. Portanto, faz-se necessário entender melhor como os fonoaudiólogos e pesquisadores conceituam o uso estrutural da CIF, particularmente os componentes de atividade e participação^(28)^ e os fatores ambientais. Reforça-se que atividades e participação foi o componente mais encontrado em análise que revisava artigos de pediatria com o uso da CIF^(23)^.

A literatura aponta^(10)^, ao investigar a percepção de familiares em casos de alterações de fala e linguagem, a importância de se considerar outros componentes para a área fonoaudiológica, dado o predomínio de análise por parte dos profissionais, quanto às funções do corpo. Nesse estudo e na elaboração do checklist, as categorias de atividades e participação foram tão consideradas quanto às de funções do corpo. Outros estudos reforçaram que entrevistas com diversos informantes e com a utilização de múltiplas ferramentas permitiu triangular os dados e privilegiar diferentes pontos de vista, para melhor compreender as crianças e os seus contextos. Os fonoaudiólogos podem considerar a incorporação da análise de todos os componentes e fatores contextuais da CIF ao avaliar e trabalhar com crianças pequenas^(10)^.

O predomínio de categorias dos componentes de funções do corpo e atividades e participação, comparativamente aos fatores ambientais, foi encontrado nesse estudo e pode ser confirmado nas pesquisas que analisaram as possibilidades de uso da CIF na saúde de crianças. Autores^(13)^ encontraram maior quantidade de estudos que abarcaram as funções corporais e atividades e participação na classificação da funcionalidade de crianças de acordo com a CIF. Também citaram a possibilidade de acompanhamento evolutivo do desenvolvimento infantil a partir da CIF, incluindo aspectos de comunicação e audição, assim como considerou-se nesse trabalho, em específico quanto aos aspectos da fala e linguagem. Os autores reforçaram a necessidade de estudos abrangentes sobre a aplicação da CIF na saúde da criança.

Em estudo anterior^(12)^ buscou-se investigar a associação de hipóteses diagnósticas fonoaudiológicas com fatores ambientais e concluiu-se pela importância de considerar tais aspectos, principalmente pela influência direta na funcionalidade da pessoa. Assim, ressalta-se que mais pesquisas deverão considerar o impacto desse componente para a área fonoaudiológica. Tais resultados encontrados podem indicar que estes aspectos não são plenamente investigados na área, conforme observa-se na baixa proporção das categorias de fatores ambientais nos dados de saúde das crianças, em serviços de saúde e educação.

A literatura^(24)^ ressalta a importância de se considerar não só os aspectos de atividade e participação, mas também os fatores contextuais, ambientais e pessoais dentro da prática fonoaudiológica, como entendimento da funcionalidade de acordo com o ambiente e as condições em que a criança se insere. Conforme autores^(29)^, o contexto interfere na funcionalidade e o conhecimento por parte do fonoaudiólogo de tais achados auxiliará no processo de seleção de indicadores base para o planejamento em saúde.

Os resultados da elaboração de um checklist de fala e linguagem indicam, com a presença de sete categorias deste componente, uma ampliação do olhar, buscando aspectos que não se limitam apenas à dimensão orgânica, assim como aqueles encontrados em literatura^(27)^. Reconhecendo-se que os fatores ambientais são importantes para a atenção à saúde da criança e do adolescente, considerando o favorecimento de atividades de linguagem no contexto social^(30)^; bem como a importância da percepção da família acerca do impacto das vivências para o desenvolvimento a criança^(10,31)^.

Com relação a elaboração de ferramentas da CIF na Fonoaudiologia, um estudo anterior^(9)^ elaborou um checklist para a reabilitação de crianças e adolescentes com deficiência auditiva. Dentre a proposta das autoras, ressaltou-se que o material ampliou a observação dos profissionais, de acordo com as necessidades do sujeito e possibilitou um acompanhamento mais individualizado do processo terapêutico.

A busca por literatura especializada mostra que são escassos os trabalhos que realizam uma análise do desenvolvimento infantil, na ausência da alteração, a partir do modelo biopsicossocial presente na CIF e consequentemente na investigação e entendimento dos seus componentes, como dados, informações e indicadores de saúde.

Assim, o checklist deste estudo pode se constituir em uma ferramenta confiável para documentar informações, em uma linguagem comum, a fim de facilitar a construção de dados de informação em saúde, acompanhamento e avaliação, além de uniformizar o conhecimento gerado, facilitar ações e políticas voltadas para a população infantil. A construção desses indicadores, de forma integrada com o usuário, abarca categorias relevantes nem sempre identificáveis em situação clínica. Ressalta-se que há necessidade de novas análises, estudos e investigações que atestem o uso da ferramenta de forma robusta e que sejam confrontados com dados de avaliação de crianças típicas e com desempenho desviante de fala e linguagem, para verificar se as categorias identificadas pelos pais e profissionais permitem caracterizar esse desenvolvimento.

A definição de categorias para o desenvolvimento típico de fala e linguagem auxilia a identificação precoce dos fatores intervenientes na aquisição de competências, facilitando a prevenção, intervenção e a comunicação multidisciplinar. O estudo e adoção futura do checklist para diferentes grupos de indivíduos e suas famílias, permitirá identificar as correlações entre as questões e a análise psicométrica no instrumento, garantindo sensibilidade e especificidade. Desta maneira, a aplicação da CIF na área fonoaudiológica estará em consonância com o desenvolvimento de ferramentas apropriadas para sua implementação na prática assistencial, preventiva, clínica e terapêutica.

Das limitações do estudo, vale ressaltar que a partir da delimitação de faixa etária (pré-escolar) desconsiderou-se categorias que poderiam ser relevantes para fala e linguagem em outras idades. Além disso, o padrão de normalidade da criança utilizado como critério de inclusão dos pais considerou apenas o corte transversal do momento do estudo, sem registro de experiência dos pais com transtornos de desenvolvimento pregressos ou atuais, considerando-se também que pais de crianças com distúrbios de comunicação podem responder de forma diferente ao questionário.

## CONCLUSÃO

Conclui-se que a partir da percepção de pais e fonoaudiólogos, foi possível selecionar as categorias da CIF mais influentes para o desenvolvimento típico de fala e linguagem. O questionário criado a partir das categorias da CIF proporcionou pontuação graduada sobre a interferência de aspectos da funcionalidade e dos fatores ambientais para a linguagem, o que permitiu a construção de um instrumento composto a partir de diferentes percepções, consequentemente sua abrangência pode ajustar-se a diferentes ações e facilitar a implementação de medidas auxiliadoras do acompanhamento na primeira infância.

A criação de um checklist, com os indicadores de desenvolvimento de fala e linguagem pela CIF, facilita o uso da Classificação e permite colocar em prática o princípio de sua construção e as interrelações entre os seus componentes. Tais achados facilitam a análise ampla e integrada do desenvolvimento típico, em seu percurso natural e individual, facilitando a identificação de alterações e a atuação precoce.
