# Supplementary material for: Checklist das categorias da CIF relevantes para o desenvolvimento de fala e linguagem
Source: Codas. 2024 Jan 22;36(2):e20220322. [Article in Portuguese] doi: 10.1590/2317-1782/20232022322en (PMC10903955; doi:10.1590/2317-1782/20232022322en)
Supplement: Translation

# Translation: Checklist of relevant ICF categories for speech and language development

**DOI:** 10.1590/2317-1782/20232022322pt

**Authors:** Fernanda Chequer de Alcântara Pinto, Ana Maria Schiefer, Jacy Perissinoto

**Affiliations:** 1 Programa de Pós-graduação em Distúrbios da Comunicação Humana, Escola Paulista de Medicina, Universidade Federal de São Paulo – UNIFESP - São Paulo (SP), Brasil.; 2 Departamento de Fonoaudiologia, Escola Paulista de Medicina, Universidade Federal de São Paulo – UNIFESP - São Paulo (SP), Brasil.

**Keywords:** International Classification of Functioning, Disability and Health Speech-Language Pathology, Health Classifications, Child Language, Child Development

## Abstract

**Purpose:**

Create a checklist of the International Classification of Functioning, Disability and Health (ICF) based on relevant categories for the development of speech and language, according to the perception of parents and speech therapists.

**Methods:**

Pilot application and research were carried out. 100 parents of preschool children with typical language/cognition development and 57 language specialist speech therapists participated in the survey. A questionnaire was created with 199 ICF categories of body function components, activities and participation, and environmental factors. Each category was scored as: indispensable (2); important (1) or unimportant (0). Statistical analysis was performed (descriptive, sum, cluster/K-means and Mann-Whitney method).

**Results:**

With the sum of the points (essential, important and unimportant) and the number of responses essential, the most relevant categories were identified for each group of respondents, as well as the set of categories in common (72 considered to have the greatest influence). The common list to the groups included the three components: body functions (30 categories/40% of the total), activities and participation (35/49.29%) and environmental factors (seven/13.20%). From the selected categories, 58.33% presented statistically significant results between the groups, regarding the relevance given.

**Conclusions:**

The categories were considered with different scores between the groups: those of body functions were more scored by speech therapists, while those of environmental factors by parents. Thus, it was possible to create a checklist from the identification of the most relevant categories for the development of speech and language, in preschool age, contemplating the components of body functions, activities and participation and environmental factors.

## INTRODUCTION

Child development, specifically language, results from neurobiological, experiential, and circumstantial components of biopsychosocial aspects^(1)^. Thus, a valuable resource has been the appreciation of procedures that consider the perception of caregivers, enlarging the view on language and functionality^(2)^.

The monitoring of child language must design instruments aimed at mapping and screening development based on normality references and deviations. For such a purpose, the International Classification of Functioning, Disability and Health (ICF) by the World Health Organization^(3)^ might help speech therapists in clinical and research practices for individualized assessment, guidance, and intervention based on the comprehensive classification proposal in different behavior domains^(4)^.

Hence, both in the scope of Research and clinic, the ICF application has been favored by tools constituted and built from different perceptions of functionality^(5)^. Its structure organized information and has been used as a statistical and epidemiological tool^(6)^ to study the functionality of healthy individuals^(7)^.

The possibilities of adopting the ICF allow the creation of new instruments, such as interviews and questionnaires^(8,9)^, especially the acknowledgments of different points of view on the same topic based on relevant aspects for the user^(10)^ and caretakers^(11)^. The ICF organization allows professionals to enlarge their view on child development, including different expectations and goals regarding the communication performance of the child in a given context^(12)^.

In this context, health professionals are responsible for developing ICF-based tools and actions that stimulate approach, practicality, and reliability in the care process^(9,13)^, in addition to showing the relationships among the categories of activities and participation, communication skills, and environmental factors^(14)^, also linked to child development^(15)^ and related assessment practices^(16)^.

Therefore, this study aimed to design a checklist for the International Classification of Functioning, Disability and Health (ICF) based on relevant categories for speech and language development, according to the perception of parents and speech therapists. Selecting certain ICF categories linked to speech and language will favor the understanding of multiple influences on communication development and performance for the processes of prevention, assessment, and intervention, thus enabling a comprehensive view and the multidisciplinary team’s communication.

## METHODS

Observational, analytical, and cross-sectional study approved by the Research Ethics Committee, protocol 1.681.979/2016. All participants and institutions signed the Informed Consent Form. Initially, a questionnaire with ICF pre-selected categories was designed, followed by the application of the instrument.

### Participants

Based on a pre-sampling calculation with a convenience sample, we interviewed 100 parents (85 mothers and 15 fathers; aged an average of 33 years old; 74% with complete high school and 26% with higher education) of children (60 boys and 40 girls), aged in average 5.16 years old (at least four years and up to five years and eleven months), with 37% between four years and four years and eleven months and 63% between five years and five years and eleven months. The sample included 57 speech therapists (language specialists certified by the Federal Council of Speech Therapy) – 98.2% female aged on average 48.61 years old.

The parents were subjected to the following inclusion criteria: more than eight years of formal education; having a child at pre-school age (between four years and five years and eleven months old); presenting no complaint regarding their child’s development; and whose child showed no (a) altered performance in the (speech therapy and neuropsychological) assessments conducted. The exclusion criterion encompassed blank answers in the questionnaire.

The speech therapists were selected according to the following inclusion criteria: working in the city of São Paulo and having the title of Language Specialist by the Federal Council of Speech Therapy. In turn, the following exclusion criteria were applied: working only with the studied age group and not answering any of the questions in the questionnaire.

### Procedures

We designed a questionnaire aimed at identifying perceptions on the typical development of speech and language in preschool children, encompassing categories to be investigated and treated by health professionals, thus ensuring early guidance and intervention. The age group was chosen based on the importance of considering child development as a continuous process that must be monitored adequately and involve the search for standardized, sensitive clinical actions for the identification of risks and alterations.

We used the International Classification of Functioning, Disability and Health (ICF)^(3)^ based on its biopsychosocial, structural model for health and related areas, considering the components of body functions and structures, activities, and participation, and environmental and personal factors. Its classification system is hierarchical, divided into chapters and categories from two to four levels^(3)^. The classification is an assessment instrument that allows selecting codes that specify the magnitude of functionality or incapacity, or still to what extent an environmental factor is an enabler or a barrier.

The questions were designed to encompass categories from three ICF components: body functions, activities, and environmental factors. The body structure component was not included since anatomical preservation was considered in research on normality^(17)^. The subdivision of ICF level two was used (362 categories in total), as indicated for clinical research and treatment evaluation^(3)^.

We removed the non-expected categories for the studied age group and those that establish the definition of situations that are not described by the ICF, reaching 199 categories transformed into questions and structured in blocks of closed questions so that the respondent indicated their position. We adopted the format of three staggered items^(18)^ with the following alternatives: Indispensable (IN) – in case of dominant agreement – two points; Important (I) – in case of dominant agreement, but not always – one point; Not important (SI) – in case of dominant disagreement – zero point.

Groups of speech therapists and parents of children were selected as respondents following the indication by the World Health Organization regarding the importance of individual participation, for ethical reasons and to ensure that the findings are more valid^(3)^.

Previously, for the questionnaire verification, we carried out the application in two groups^(19)^ of characterization like the sampling groups. In this context, each individual was instructed to evaluate the language and instrument used. It is worth highlighting that no changes occurred after the procedure.

Two groups answered the questionnaire containing 199 questions. For the application in the group of professionals, the categories were introduced with the same ICF description. The respondents should answer on the importance of each category for developing speech and language at pre-school age. As to the group of parents, the ICF categories were summarized and made more objective so as not to lose their definitions and to favor understanding. Each individual was instructed to reflect on their preschool child and answer on the importance of that category for developing speech and language.

The collection was carried out in a school of early childhood education, located in the south area of the city of São Paulo. To ensure participation, parents answered an initial survey on the child's development. Subsequently, the assessment process involved the application of a hearing screening, assessment of receptive and expressive language, and a neuropsychological test (with the help of a psychologist) to measure the non-verbal intelligence quotient para. All procedures were performed in the school environment.

The assessment process investigated the cochlear-palpebral reflex (CPR), in which the child was presented with a high-intensity, short-duration sound stimulus, which provides data on the child's overall maturational development. In terms of language, the Assessment of Language Development (ADL) was used^(20)^, a scale that aims to assess the receptive and expressive domains of language, in the age range of one year to six years and eleven months. In the neuropsychological assessment, the non-verbal intelligence quotient (IQ) was measured using the Son-R 2 ½-7 [a]^(21)^, which includes the subtests: mosaics, patterns, categories, and situations, forming an execution, and reasoning scale.

Out of the total of children enrolled in the school, 134 (corresponding to 86.54% of the total of school children in the institution) were authorized by their parents to be evaluated. Out of these, 117 children presented adequate results in the tests (87.31%) and 17 of them had altered results, being excluded from the sample and referred to diagnosis service (12.69%).

Following the children’s assessment, groups of up to ten parents were called in turn for the assessment feedback (up to one hour long). During the meeting and after a brief explanation in the ICF, the parents were instructed to answer the questionnaire by scoring the degree of influence of each category on their child’s development of speech and language. In total, 106 parents answered the questionnaire, out of which six were excluded randomly to reach the 100 pre-defined individuals.

The group of speech therapists was composed of speech therapists working in the city of São Paulo with a language specialist title validated by the Federal Council of Speech Therapy (CFFa). Each speech therapist kept the questionnaire containing a brief explanation of ICF, individually, for up to ninety days.

### Statistical analysis

The findings were tabulated, and the answers were analyzed. To learn which questions were more relevant, we calculated the following items: the sum of scores by a question – corresponding to zero, one, or two –, characterizing the SOMA analysis criterion; counting of indispensable answers – TOTAL2 criterion. The classification was established involving a cluster analysis (K-means method) by criterion (SOMA and TOTAL2) and group (professionals and parents), defining five groups in decreasing order: 1 (higher score), 2, 3, 4, and 5 (lower scores). Mann-Whitney non-parametric test was used to compare the answers between the groups, with a significance level of 5% and a confidence interval of 95%.

## RESULTS

It is worth highlighting that all categories investigated received some score, which confirms that all questions were answered. Among the ten best-scored categories, the parents considered the nuclear family (e310), the component of environmental factors, the most relevant for speech and language development. For the speech therapists, in turn, the first category was the component of activities and participation, language acquisition (d132).

For the group of parents, the activity category of Listening (d115) appeared in the top ten, while all others were linked to the component of body functions, especially regarding mental function, and cardiovascular and respiratory systems. For speech therapists, the most relevant categories were divided among body functions, especially regarding mental functions, voice, and speech, while activities and participation were linked to learning and knowledge application.

The cluster was designed based on the results identified by the SOMA and TOTAL2 criteria, detecting the best-scored categories for the checklist of typical speech and language development. Table 1 compares the selection of categories, among the most relevant ones by group, based on the first three groups. According to the pre-defined criteria, we decided to disregard the questions from the last two groups (4 and 5), referring to the categories with the lowest scores defined by the groups.

**Table 1:** Final cluster selection for both groups

**Question/ Category**	**Parents Cluster**	**Phono Cluster**	**Final Checklist**
Functions of consciousness (b110)	2	1	1
Orientation functions (b114)	2	1	1
Intellectual functions (b117)	1	1	1
Global psychosocial functions (b122)	2	1	1
Temperament and personality functions (b126)	3	3	1
Energy and impulse functions (b130)	3	3	1
Sleep functions (b134)	3	3	1
Attention functions (b140)	2	1	1
Memory functions (b144)	2	1	1
Psychomotor functions (b147)	2	2	1
Emotional functions (b152)	3	2	1
Perception functions (b156)	1	1	1
Thought functions (b160)	2	2	1
Higher cognitive functions (b164)	3	1	1
Mental functions of language (b167)	2	1	1
Mental functions of sequencing complex movements (b176)	3	2	1
Experience of self and time functions (b180)	3	2	1
Seeing functions (b210)	2	3	1
Hearing functions (b230)	2	1	1
Vestibular functions (b235)	3	3	1
Proprioceptive functions (b260)	3	3	1
Voice functions (b310)	2	1	1
Articulation functions (b320)	2	1	1
Fluency and rhythm of speech functions (b330)	2	1	1
Alternative vocalization functions (b340)	3	2	1
Respiratory functions (b440)	1	3	1
Respiratory muscle functions (b445)	1	3	1
Ingestion functions (b510)	1	3	1
Muscle power functions (b730)	3	3	1
Control of voluntary movements (b760)	3	3	1
Observing (d110)	2	3	1
Listening (d115)	1	1	1
Other purposeful sensing (d120)	2	3	1
Copying (d130)	3	1	1
Language acquisition (d132)	3	1	1
Rehearsing (d135)	3	2	1
Acquiring concepts (d137)	3	1	1
Acquiring information (d138)	2	1	1
Learning to read (d140)	2	3	1
Learning to write (d145)	2	3	1
Acquiring skills (d155)	3	2	1
Focusing attention (d160)	2	2	1
Thinking (d163)	3	2	1
Decision-making (d177)	3	3	1
Performing a single task (d210)	3	3	1
Carrying out daily routine (d230)	3	3	1
Communicating – receiving spoken messages (d310)	2	2	1
Communicating – receiving non-verbal messages (d315)	3	2	1
Communicating – comprehending messages in formal sign language (d320)	3	2	1
Speech (d330)	2	1	1
Singing (d332)	2	2	1
Non-verbal messages production (d335)	3	2	1
Conversation (d350)	3	1	1
Eating (d550)	2	3	1
Drinking (d560)	2	3	1
Basic interpersonal interactions (d710)	3	2	1
Complex interpersonal interactions (d720)	3	3	1
Formal relationships (d740)	2	3	1
Informal social relationships (d750)	3	3	1
Family relationships (d760)	2	2	1
Informal education (d810)	3	3	1
Early childhood education (d815)	1	2	1
School education (d820)	1	2	1
Recreation and leisure (d920)	3	2	1
Human rights (d940)	3	3	1
Sound (e250)	3	2	1
Nuclear family (e310)	1	2	1
Extended family (e315)	2	3	1
Friends (e320)	3	3	1
Health professionals (e355)	2	3	1
Other professionals (e360)	3	3	1
Health services, systems, and policies (e580)	2	3	1

Note: Analysis by cluster

Chart 1 shows the checklist designed based on the indication by both parents and professionals on the most relevant categories for the development of speech and language according to the ICF.

**Chart 1:** ICF Checklist for speech and language development in preschoolers

**ICF CHECKLIST FOR SPEECH AND LANGUAGE DEVELOPMENT IN PRESCHOOLERS**
**Body functions component**
Functions of consciousness (b110) – general mental functions of alertness and consciousness, including clarity and continuity of wakefulness.
Orientation functions (b114) – general mental functions linked to the knowledge and determination of a person's relationship with themselves, with other people, with objects, and with space.
Intellectual functions (b117) – general mental functions required to understand and constructively integrate the different mental functions, including all cognitive functions and their development throughout life.
Global psychosocial functions (b122) – general mental functions, as they develop throughout life, which are necessary for understanding and constructively integrating the mental functions that lead to the formation of the interpersonal skills needed to establish reciprocal social interactions, both in terms of meaning and purpose.
Temperament and personality functions (b126) – general mental functions linked to a temperament that makes the individual react in a certain way to situations, including the set of mental characteristics that differentiate that individual from other people.
Energy and impulse functions (b130) – general mental functions of the physiological and psychological mechanisms that stimulate the individual to act persistently to satisfy their specific needs and objectives.
Sleep functions (b134) – general mental functions of physical and mental disconnection from the immediate environment of a periodic, reversible, and selective nature, accompanied by characteristic physiological changes.
Attention functions (b140) – specific mental functions of concentrating on an external stimulus or internal experience for the required length of time.
Memory functions (b144) – specific mental functions for recording and storing information and retrieving it when necessary.
Psychomotor functions (b147) – specific mental functions for controlling motor and psychological events at a bodily level.
Emotional functions (b152) – specific mental functions linked to feelings and the affective components of mental processes.
Perception functions ((b156) – specific mental functions linked to the recognition and interpretation of sensory stimuli.
Thought functions (b160) – specific mental functions linked to the ideational component of the mind.
Higher cognitive functions (b164) – specific mental functions especially dependent on the frontal lobes of the brain, including complex goal-directed behaviors such as decision-making, abstract thinking, planning, and execution of plans, mental flexibility, and deciding what behaviors are appropriate in specific circumstances, often called executive functions.
Mental functions of language (b167) – specific mental functions for recognizing and using signs, symbols, and other components of a language.
Mental functions of sequencing complex movements (b176) – specific mental functions for sequencing and coordinating complex, purposeful movements.
Experience of self and time functions (b180) – specific mental functions linked to awareness of one's own identity, one's own body, one's posture in one's environment, and in time.
Seeing functions (b210) – sensory functions linked to the perception of light and the shape, size, form, and color of a visual stimulus.
Hearing functions (b230) – sensory functions that allow us to perceive sounds and discriminate their location, intensity, loudness, and quality.
Vestibular functions (b235) – sensory functions of the inner ear linked to position, balance, and movement.
Proprioceptive functions (b260) – sensory functions that allow us to feel the relative position of body parts.
Voice functions (b310) – functions of the production of various sounds by the passage of air through the larynx.
Articulation functions (b320) – the function of producing speech sounds.
Fluency and rhythm of speech functions (b330) – production function of the flow and rhythm of speech.
Alternative vocalization functions (b340) – functions of producing other forms of vocalization.
Respiratory functions (b440) – functions linked to the inhalation of air into the lungs, the exchange of gases between air and blood, and the expulsion of air.
Respiratory muscle functions (b445) – functions of the muscles involved in breathing.
Ingestion functions (b510) – functions linked to the ingestion and manipulation of solids or liquids in the body through the mouth.
Muscle power functions (b730) – functions linked to the force generated by the contraction of a muscle or group of muscles.
Control of voluntary movements (b760) – Control of voluntary movements.
**Activities and participation component**
Observing (d110) – intentionally using the sense of sight to experience visual stimuli, such as visually following or tracking an object, watching a sporting event, or observing people or children playing.
Listening (d115) – intentionally using the sense of hearing to experience auditory stimuli, such as listening to the radio, human voices, music, a lesson, or the telling of a story.
Other purposeful sensing (d120) – intentionally using the body's other basic senses to experience stimuli, such as touching or feeling textures, tasting sweets, or smelling flowers.
Copying (d131) – copying or mirroring as a basic component of learning, such as copying a facial expression, a gesture, a sound, or the letters of an alphabet.
Language acquisition (d132) – developing the ability to represent people, objects, events, and feelings through words, symbols, phrases, and sentences.
Rehearsing (d135) – repeating a sequence of events or symbols as a basic component of learning, such as counting in tens or practicing reciting a rhyme with gestures or chords on a musical instrument.
Acquiring concepts (d137) – developing the ability to understand and use basic and complex concepts linked to the characteristics of things, people, or events.
Acquiring information (d138) – obtaining facts about people, things, and events, such as asking why, what, where, and how, and asking for names.
Learning to read (d140) – developing the ability to read printed material (including Braille and other symbols) fluently and accurately, such as recognizing characters and letters of the alphabet, vocalizing written words with the correct pronunciation, and understanding written words of sentences.
Learning to write (d145) – developing the ability to produce symbols that represent sounds, words, or sentences that have meaning (including Braille writing and other symbols), how to write efficiently, and use correct grammar.
Acquiring skills (d155) – developing basic and complex skills in using an integrated set of actions or tasks to initiate and complete the acquisition of a skill, such as manipulating tools or toys, or playing games.
Focusing attention (d160) – intentionally focusing on a specific stimulus, disconnecting from distracting noise.
Thinking (d163) – formulating and manipulating ideas, concepts, and images, whether or not they are aimed at a goal, alone or with others, such as creating fiction, proving a theorem, playing with ideas, debating ideas, meditating, pondering, speculating, or reflecting.
Decision-making (d177) – choosing between options, implementing the choice, and evaluating the effects of the choice, such as selecting and purchasing a specific item or deciding to put into practice and carry out a task among several tasks that need to be done.
Performing a single task (d210) – performing simple or complex coordinated actions linked to the mental and physical components of a single task, such as initiating a task, organizing the time, space, and materials for a task, regulating the performance of the task and executing, completing, and maintaining the task.
Carrying out daily routine (d230) – performing and coordinating simple or complex actions to plan, manage, and complete the requirements of day-to-day procedures or duties, such as managing time and making plans for various activities throughout the day.
Communicating – receiving spoken messages (d310) – understanding the literal and implicit meanings of messages in spoken language, such as distinguishing whether a sentence has a literal meaning or is an idiomatic expression.
Communicating – receiving non-verbal messages (d315) – understanding the literal and implicit meanings of messages conveyed by gestures, symbols, and drawings, such as realizing that a child is tired when they rub their eyes or that an alarm means there is a fire.
Communicating – comprehending messages in formal sign language (d320) – receiving and understanding messages in standard sign language with literal and implicit meaning.
Speech (d330) – producing words, phrases, and longer passages in spoken messages with literal and implicit meaning, such as expressing a fact or telling a story in oral language.
Singing (d332) – using tones in a sequence that results in a melody to convey messages.
Non-verbal message production (d335) – using gestures, symbols, and drawings to convey messages, such as shaking the head to indicate disagreement or drawing a figure or diagram to convey a complex factor or idea.
Conversation (d350) – initiating, maintaining, and concluding an exchange of thoughts and ideas, using written, oral, sign, or other forms of language, with one or more acquaintances or strangers, in a formal or informal setting.
Eating (d550) – performing the coordinated tasks and actions of eating the food served, bringing it to the mouth and consuming it in a naturally acceptable manner; cutting or breaking the food into pieces; opening packages and packets; using eating utensils; activities related to meals, banquets, and dinners.
Drinking (d560) – picking up the drink, bringing it to the mouth, and consuming the drink in a culturally acceptable way, mixing, months and pouring liquids for drinking, opening bottles and cans, drinking through a straw or drinking running water from the tap or a fountain; breastfeeding.
Basic interpersonal interactions (d710) – interacting with people in a contextually and socially appropriate way, such as showing consideration and esteem when appropriate or reacting to the feelings of others.
Complex interpersonal interactions (d720) – maintaining and controlling interactions with other people in a contextually and socially appropriate way, such as controlling emotions and impulses, controlling verbal and physical aggression, acting independently in social interactions, and following social rules and conventions when, for example, playing, studying, or working with other people.
Formal relationships (d740) – creating and maintaining specific relationships in formal environments, such as with teachers, employees, professionals, or service providers.
Informal social relationships (d750) – initiating relationships with others, such as causal relationships with people who live in the same community or residence, or with co-workers, students, leisure companions, or people with similar backgrounds or professions.
Family relationships (d760) – creating and maintaining kinship relationships, such as with members of the family nucleus, relatives, adoptive and foster families, and non-consanguineous relatives, more distant relationships such as second cousins or legal guardians.
Informal education (d810) – learning at home or in another non-institutional environment, such as learning crafts and other skills as parents or family members, or homeschooling.
Early childhood education (d815) – learning at an initial level of organized instruction, designed primarily to introduce the child to the school environment and prepare them for compulsory education, such as acquiring skills in a nursery or similar setting in preparation for school.
School education (d820) – gaining access to the school, participating in all school-related responsibilities and privileges, and learning the course material, subject matter, and other curricular requirements in an elementary and secondary educational program, including attending school regularly, working cooperatively with other students, following teachers' directions, organizing, studying and completing assigned tasks and projects, and progressing to the other stages of education.
Recreation and leisure (d920) – participating in any form of play, recreational or leisure activity, such as informal or organized play or sports, a physical exercise program, relaxation, entertainment, going to art galleries, museums, cinema or theater, taking part in crafts or hobbies, reading for pleasure, playing musical instruments, going on excursions, sightseeing or traveling for pleasure.
Human rights (d940) – enjoying all the nationally and internationally recognized rights that are attributed to people by the mere fact of their human condition, such as the human rights recognized by the United Nations in the Universal Declaration of Human Rights (1948), the Convention on the Rights of the Child (1989), the Standards on Equal Opportunities for Persons with Disabilities (1993), and the Convention on the Rights of Persons with Disabilities (2006); the right to self-determination or autonomy; and the right to control one's destiny.
**Environmental factors component**
Sound (e250) – a phenomenon that is or can be heard, such as knocking, tapping, banging, singing, whistling, shouting, or buzzing, in any volume, timbre, or pitch, and which can provide useful information about the world.
Nuclear family (e310) – individuals related by birth, marriage, or other relationships recognized by cultures, such as nuclear family, spouses, partners, parents, siblings, children, foster parents, adoptive parents, and grandparents.
Extended family (e315) – individuals related through family or marriage, or other relationships recognized by culture, such as relatives, aunts, uncles, nephews, and nieces.
Friends (e320) – individuals who are close and continuous in relationships characterized by mutual trust and support.
Health professionals (e355) – all service providers working in the context of the healthcare system, such as doctors, nurses, physiotherapists, occupational therapists, speech therapists, audiologists, prosthetists, and medical social workers.
Other professionals (e360) – all service providers who work outside the health system but provide health-related services, such as social workers, teachers, architects, or designers.
Health services, systems, and policies (e580) – services, systems, and policies for the prevention and treatment of health problems, the provision of medical rehabilitation, and the promotion of a healthy lifestyle.
**Body structures component (something to declare):**
**Personal factors component (description):**

Source: elaborated by the authors, 2023

The checklist defined herein included 72 categories. Out of these, regarding body functions, 30 were present according to the ICF subdivision by chapters: 17 for mental functions; four for sensory functions and pain; four for voice functions and speech; two for functions of the cardiovascular, hematologic, immunologic, and respiratory systems; one for functions of the digestive, metabolic, and endocrine systems; two for neuromusculoskeletal functions related to movements.

As to activities and participation, 35 categories appeared as more relevant: 14 for learning and knowledge application, two for general tasks and demands, 7 for communicating, two for personal care, five for interpersonal relationships and interactions, three for life areas, and two for community, social, and civil life.

As to the environmental factors, seven categories belonged to the component for three chapters: natural environmental and man-made environmental changes; support and relationships; services, systems, and policies.

The comparative analysis between the answers of both groups (Table 2) showed that 42 categories (58.33%) had significant intergroup results regarding the difference in the given relevance, including 21 for body functions (50%); 18 for activities and participation (42.85%), and three for environmental factors (7.14%).

**Table 2:** Comparing the answers between parents and speech therapists

**CATEGORY**	**PARENT GROUP MEAN SUM**	**SPEECH THERAPISTS MEAN SUM**	**MANN-WHITNEY TEST (p)**	**RESULT**
Functions of consciousness (b110)	1.37	1.65	0.001*	Parents < Professionals
Orientation functions (b114)	1.32	1.61	0.001*	Parents < Professionals
Intellectual functions (b117)	1.54	1.74	0.015*	Parents < Professionals
Global psychosocial functions (b122)	1.32	1.77	<0.001*	Parents < Professionals
Temperament and personality functions (b126)	1.22	1.05	0.111	Parents = Professionals
Energy and impulse functions (b130)	1.22	1.04	0.080	Parents = Professionals
Sleep functions (b134)	1.18	1.07	0.308	Parents = Professionals
Attention functions (b140)	1.28	1.58	0.001*	Parents < Professionals
Memory functions (b144)	1.33	1.72	<0.001*	Parents < Professionals
Psychomotor functions (b147)	1.27	1.25	0.967	Parents = Professionals
Emotional functions (b152)	1.22	1.37	0.073	Parents = Professionals
Perception functions (b156)	1.44	1.63	0.032*	Parents < Professionals
Thought functions (b160)	1.29	1.47	0.034*	Parents < Professionals
Higher cognitive functions (b164)	1.20	1.61	<0.001*	Parents < Professionals
Mental functions of language (b167)	1.34	1.82	<0.001*	Parents < Professionals
Mental functions of sequencing complex movements (b176)	1.18	1.21	0.600	Parents = Professionals
Experience of self and time functions (b180)	1.19	1.37	0.033*	Parents < Professionals
Seeing functions (b210)	1.33	0.95	<0.001*	Parents < Professionals
Hearing functions (b230)	1.31	1.67	<0.001*	Parents < Professionals
Vestibular functions (b235)	1.17	1.00	0.076	Parents = Professionals
Proprioceptive functions (b260)	1.18	1.16	0.969	Parents = Professionals
Voice functions (b310)	1.29	1.54	0.003*	Parents < Professionals
Articulation functions (b320)	1.30	1.74	<0.001*	Parents < Professionals
Fluency and rhythm of speech functions (b330)	1.29	1.67	<0.001*	Parents < Professionals
Alternative vocalization functions (b340)	1.16	1.33	0.029*	Parents < Professionals
Respiratory functions (b440)	1.48	1.12	0.001*	Parents > Professionals
Respiratory muscle functions (b445)	1.47	1.09	0.001*	Parents > Professionals
Ingestion functions (b510)	1.39	0.89	<0.001*	Parents > Professionals
Muscle power functions (b730)	1.25	1.05	0.032*	Parents > Professionals
Control of voluntary movements (b760)	1.24	1.21	0.838	Parents = Professionals
Observing (d110)	1.37	1.21	0.088	Parents = Professionals
Listening (d115)	1.47	1.68	0.009*	Parents < Professionals
Other purposeful sensing (d120)	1.33	1.02	0.001*	Parents > Professionals
Copying (d130)	1.21	1.68	<0.001*	Parents < Professionals
Language acquisition (d132)	1.16	1.89	<0.001*	Parents < Professionals
Rehearsing (d135)	1.19	1.42	0.007*	Parents < Professionals
Acquiring concepts (d137)	1.17	1.68	<0.001*	Parents < Professionals
Acquiring information (d138)	1.29	1.77	<0.001*	Parents < Professionals
Learning to read (d140)	1.35	0.81	<0.001*	Parents > Professionals
Learning to write (d145)	1.34	0.79	<0.001*	Parents > Professionals
Acquiring skills (d155)	1.23	1.28	0.437	Parents = Professionals
Focusing attention (d160)	1.28	1.47	0.056	Parents = Professionals
Thinking (d163)	1.21	1.49	0.003*	Parents < Professionals
Decision-making (d177)	1.23	1.04	0.043*	Parents > Professionals
Performing a single task (d210)	1.18	1.05	0.182	Parents = Professionals
Carrying out daily routine (d230)	1.16	1.05	0.301	Parents = Professionals
Communication – receiving spoken messages (d310)	1.29	1.39	0.228	Parents = Professionals
Communication – receiving non-verbal messages (d315)	1.23	1.39	0.053	Parents = Professionals
Communication–comprehending messages in formal sign language (d320)	1.14	1.14	0.738	Parents = Professionals
Speak (d330)	1.33	1.60	0.004*	Parents < Professionals
Singing (d332)	1.28	1.33	0.528	Parents = Professionals
Non-verbal messages production (d335)	1.18	1.42	0.004*	Parents < Professionals
Conversation (d350)	1.23	1.51	0.001*	Parents < Professionals
Eating (d550)	1.26	0.88	0.001*	Parents > Professionals
Drinking (d560)	1.29	0.89	0.001*	Parents > Professionals
Basic interpersonal interactions (d710)	1.25	1.42	0.039*	Parents > Professionals
Complex interpersonal interactions (d720)	1.21	1.14	0.531	Parents > Professionals
Formal relationships (d740)	1.34	0.98	<0.001*	Parents > Professionals
Informal social relationships (d750)	1.16	1.05	0.276	Parents = Professionals
Family relationships (d760)	1.26	1.19	0.698	Parents > Professionals
Informal education (d810)	1.21	1.18	0.672	Parents > Professionals
Early childhood education (d815)	1.41	1.35	0.658	Parents = Professionals
School education (d820)	1.41	1.35	0.885	Parents = Professionals
Recreation and leisure (d920)	1.25	1.23	0.998	Parents = Professionals
Human rights (d940)	1.20	1.07	0.338	Parents = Professionals
Sound (e250)	1.14	1.26	0.114	Parents = Professionals
Nuclear family (e310)	1.54	1.42	0.160	Parents = Professionals
Extended family (e315)	1.26	1.05	0.032*	Parents > Professionals
Friends (e320)	1.21	1.23	0.929	Parents = Professionals
Health professionals (e355)	1.31	1.16	0.172	Parents = Professionals
Other professionals (e360)	1.21	0.96	0.025*	Parents > Professionals
Health services, systems, and policies (e580)	1.32	1.02	0.012*	Parents > Professionals

Note: Mann-Whitney Test

* P-values marked (*) indicate a statistically significant result

For the body functions component, the speech therapists had a higher score for the SOMA variable than the parents in 18 (60%) out of the 30 categories analyzed for the checklist. As to activities and participation, the speech therapists had a higher score in 16 (45.71%) out of the 35 categories analyzed. Finally, for the component of environmental factors, the speech therapists had a higher score for the SOMA variable than the group of parents in two (28.57%) out of the seven selected categories.

## DISCUSSION

This study aimed to design a checklist based on the aspects of speech and language functionality. The use of this instrument seeks to favor the comprehensiveness of the professional view by allowing the interactions and impacts for the child to acquire and develop both speech and language. It is worth highlighting that the use of the ICF as a structural basis favors the selection and formalization of categories, standardizing the nomenclature used and allowing comparing data and confirming the possibility of designing other assessment instruments, screening, and/or development monitoring in the scope^(22)^.

A systematic review analyzing the use of the ICF in Brazilian pediatrics indicated that 40% of the studies used classification checklists in their design^(23)^, despite indicating an incipient use for children by health professionals. The authors reinforced the importance of its use for the definition and planning of resources, services, and policies, by organizing information, favoring communication, and seeking more holistic approaches.

Designing normality-based instruments adjusts and comprehends relevant aspects according to the age group, allowing professionals to enlarge their view on health and identifying behavior and situations that are not typical. Such a process favors decision-making and the follow-up and monitoring of children, as well as characterizes more individually the functionality demands of the population. However, as reinforced by authors^(7,23)^, there are few surveys addressing populations with no health alterations in the scope of prevention and promotion using the ICF, even though the WHO foresees the insertion of classification in populations, regardless of the presence of a clinical condition^(3).^

The use of the ICF allows learning the relationships present in the components of child development, favoring investigation and considering important aspects of the subject’s social and interactive sharing beyond body functioning. The importance of screening and monitoring individuals throughout their development trajectory is reinforced in a prior study indicating the use of the classification to organize and standardize information on functionality. The authors reinforce that the design of ICF-related instruments, such as checklists, is fundamental for the use of the biopsychosocial model in clinical practice, in services encompassing hearing screening, monitoring of language development, and therapeutic evolution^(8)^.

The speech therapy practice recommends and encourages the use of the International Classification of Functioning, Disability and Health (ICF) structure. The thematic analysis in a scoping review showed that the ICF was applied in the assessment, intervention, and supply of health services. However, some gaps appeared regarding the use of ICF-based tools in the speech therapy clinical practice and its social understanding, whose use favors a more holistic understanding of functionality, incapacity, and evidence-based clinical decisions^(16)^.

An integrative literature analysis^(24)^ on ICF use revealed its benefits since the anamnesis process to child language development, including the use of questionnaires that simplify the classification, as well as the design of tools based on its assumptions for language acquisition and development. Such a scenario reinforces the importance of designing a checklist targeted at child language development, as structured herein.

The development of action skills based on a vast variety of data collection methods and documentation tools helps speech therapists communicate accurately the results from services rendered, thus improving the clinical decision-making process^(25)^. Such a scenario directly impacts the understanding of the action dimension for the speech therapy field and the need for a greater focus on the aspects of functionality, especially those linked to communication^(14)^.

To outline these findings, the perception of expert professionals is fundamental, as well as the subjects, who know the impacts on functionality and incapacity that are experienced. The use of the ICF allowed learning the relevant categories for typical speech and language development, encompassing body functions components, activities, participation, and environmental factors, as well as other studies demonstrating the applicability of the ICF as guiding principles of health services^(9,13,23,26)^.

Among the most relevant categories pointed out by the groups were especially those linked to the component of body function, which corroborates other studies^(27)^. Such findings have demonstrated that physiological elements are very relevant for child development, which reinforces the structure of professional training and the dissemination of health knowledge in the biomedical model. Thereby, speech therapists and researchers must better understand the concept of the ICF structural use, particularly the components of activity and participation^(28)^ and environmental factors. It is worth highlighting that the activities and participation component was the most frequent in analysis reviewing pediatrics articles using the ICF^(23)^.

By investigating the perception of family members in cases of speech and language alterations, the literature points out^(10)^ the importance of considering other components for the speech therapy field given the predominance of analysis by professionals regarding body functions. Herein and in the design of the checklist, the categories of activities and participation were considered as body functions. Other studies have reinforced that interviews with several informants using multiple tools allowed screening of the data and favored different points of view for a better understanding of children and their contexts. Speech therapists might consider incorporating the analysis of all components and context factors of the ICF by evaluating and working with small children^(10)^.

Our study observed the predominance of categories of the body functions and activities and participation components, compared with the environmental factors, which is corroborated in studies analyzing possibilities of ICF use in child health. We^(13)^ have found a greater number of studies addressing body functions and activities and participation in the classification of children’s functionality, according to the ICF. They have also mentioned the possibility of monitoring child development based on the ICF, including communication and hearing aspects, as considered herein, specifically regarding speech and language elements. We also highlight that more comprehensive studies should be conducted on the ICF application in child health care.

A prior study^(12)^ sought to investigate the association of speech therapy diagnosis hypotheses including environmental factors, demonstrating the importance of considering such aspects, especially by their direct influence on the subject’s functionality. Thus, it is worth highlighting that further research should consider the impact of this component on the speech therapy field. Such results might indicate that these aspects are yet to be fully investigated in the area, as shown in the low proportion of categories of environmental factors in child health data for health and education services.

The literature^(24)^ highlights the importance of considering not only the aspects of activity and participation, but also context, environmental, and personal factors in the speech therapy practice, such as by understanding functionality according to the environment as the conditions of the child. According to some authors^(29)^, context interferes with functionality; therefore, the speech therapist's knowledge of such findings might benefit the selection process of base indicators for health planning.

With the presence of seven categories from this component, the results from a checklist for speech and language indicate a more comprehensive view by seeking aspects that are not limited to the organic dimension, as well as those found in the literature^(27)^. The environmental factors proved important for the health care of children and adolescents considering that language activities are favored in the social context^(30)^. It is also worth highlighting the importance of the family's perception of the impact of these experiences on the child's development^(10,31)^.

As to the design of ICF tools for speech therapy, a prior study^(9)^ developed a checklist for the recovery of children and adolescents with hearing impairment. The authors highlighted that the material has enlarged professional views regarding individual needs and allowed for more personal monitoring of the therapeutic process.

The specialized literature reports only a few studies analyzing child development based on the absence of alterations through the biopsychosocial model from the ICF, hence the investigation and understanding of its components, such as data, information, and health indicators.

Thereby, the checklist herein might represent a reliable tool to document information in an accessible language to favor the collection of health information data, monitoring, and assessment, in addition to standardizing the knowledge generated and enabling actions and policies targeted to the child population. The design of these indicators integrated with the user encompasses relevant categories that are not always identifiable in a clinical situation. It is worth highlighting the need for further analyses, studies, and investigations that confirm the robust use of the tool against assessment data of both typical children and those with deviant speech and language performance to learn whether the categories indicated by parents and professionals allow characterizing such development.

Defining categories for the typical development of speech and language favors the early detection of factors that interfere with the acquisition of skills, benefiting prevention, intervention, and multidisciplinary communication. Both the study and further use of the checklist for different groups of individuals and their families will allow for identifying correlations between the questions and the psychometric analysis from the instrument, thus ensuring sensitivity and specificity. Thereby, the ICF application in the speech therapy area is in line with the design of adequate tools for its implementation in care, preventive, clinical, and therapeutic practice.

As to the study limitations, it is worth highlighting that the age group (pre-school) delimitation disregarded potentially relevant categories for speech and language at other ages. In addition, the child normality pattern used as inclusion criteria for the parents considered only the cross-sectional design at the time of the study, with no record of the parents’ experience with previous or current developmental disorders, also considering that parents of children with communication disorders may respond differently to the questionnaire.

## CONCLUSION

The perception of both parents and speech therapists allowed us to select the ICF categories that most influence the typical development of speech and language. The questionnaire designed based on the ICF categories provided a graded scoring on the interference of functionality aspects and environmental factors for language, which allowed us to elaborate on an instrument composed of different perceptions. Hence, its comprehensiveness might adjust to different actions and favor the implementation of measures that benefit early childhood care.

Designing a checklist introducing the development indicator of speech and language based on the ICF favors the use of classification and allows putting into practice its structural principle and the inter-relationships of its components. These findings benefit a comprehensive and integrated analysis of the natural and individual trajectory of typical development, thus enabling detection of alterations and the start of early intervention.
